# EHPnet: Design for the Environment Partnerships

**Published:** 2008-05

**Authors:** Erin E. Dooley

**http://www.epa.gov/oppt/dfe/pubs/projects/index.htm**

The U.S. Environmental Protection Agency (EPA) Design for the Environment Program works with various industries to minimize the use of toxic chemicals and identify suitable replacement for such ingredients. As part of this program, the EPA convenes partnerships around specific industries to evaluate the health considerations, performance, and costs of traditional and alternative technologies, materials, and processes used in those industries. Two of these partnerships, described on the program’s Partnership Projects website, seek to identify environmentally sound alternatives to flame retardants currently in use.

Each partnership’s website provides an overview of the partnership and the chemicals it focuses on. The Flame Retardants in Printed Circuit Boards Partnership consists of members from government, the computer and chemical industries, and environmental advocacy organizations. This partnership is just beginning its third year and, according to the website, plans this spring to publish a report on its findings to date.

The Furniture Flame Retardancy Partnership began in 2003 and convenes government, the furniture and chemical industries, and environmental groups. Its site describes current regulatory actions in the area of flame retardancy and highlights its latest publication, Environmental Profiles of Chemical Flame-Retardant Alternatives for Low-Density Polyurethane Foam. This two-volume overview of alternatives to the compound pentaBDE includes detailed hazard assessments of more than 10 chemicals believed to be viable substitutes.

The Furniture Partnership page offers background information on green chemistry and green engineering as well as a chart of international and national milestones relevant to the decision to ban penta-BDE. Visitors can also download a pamphlet on environmentally preferable methods for achieving furniture fire safety standards, a fact sheet on flame-retardant alternatives for foam used for furniture manufacturing, and the Environmental Profiles report.

